# WHO Grade Loses Its Prognostic Value in Molecularly Defined Diffuse Lower-Grade Gliomas

**DOI:** 10.3389/fonc.2021.803975

**Published:** 2022-01-10

**Authors:** Louise Carstam, Alba Corell, Anja Smits, Anna Dénes, Hanna Barchéus, Klara Modin, Helene Sjögren, Sandra Ferreyra Vega, Thomas Olsson Bontell, Helena Carén, Asgeir Store Jakola

**Affiliations:** ^1^ Department of Neurosurgery, Sahlgrenska University Hospital, Gothenburg, Sweden; ^2^ Institute of Neuroscience and Physiology, Sahlgrenska Academy, University of Gothenburg, Gothenburg, Sweden; ^3^ Department of Neurology, Sahlgrenska University Hospital, Gothenburg, Sweden; ^4^ Clinical Genetics and Genomics, Sahlgrenska University Hospital, Gothenburg, Sweden; ^5^ Sahlgrenska Center for Cancer Research, Department of Laboratory Medicine, Institute of Biomedicine, Sahlgrenska Academy, University of Gothenburg, Gothenburg, Sweden; ^6^ Department of Clinical Pathology and Cytology, Sahlgrenska University Hospital, Gothenburg, Sweden; ^7^ Department of Neurosurgery, St. Olavs University Hospital, Trondheim, Norway

**Keywords:** lower-grade glioma, prognostic factors, WHO grade, *IDH*-mut, *CDKN2A/B* deletion, astrocytoma, oligodendroglioma, extent of resection

## Abstract

**Background:**

While molecular insights to diffuse lower-grade glioma (dLGG) have improved the basis for prognostication, most established clinical prognostic factors come from the pre-molecular era. For instance, WHO grade as a predictor for survival in dLGG with *isocitrate dehydrogenase* (*IDH*) mutation has recently been questioned. We studied the prognostic role of WHO grade in molecularly defined subgroups and evaluated earlier used prognostic factors in the current molecular setting.

**Material and Methods:**

A total of 253 adults with morphological dLGG, consecutively included between 2007 and 2018, were assessed. *IDH* mutations, codeletion of chromosomal arms 1p/19q, and *cyclin-dependent kinase inhibitor 2A/B (CDKN2A/B)* deletions were analyzed.

**Results:**

There was no survival benefit for patients with WHO grade 2 over grade 3 *IDH*-mut dLGG after exclusion of tumors with known *CDKN2A/B* homozygous deletion (n=157) (log-rank p=0.97). This was true also after stratification for oncological postoperative treatment and when astrocytomas and oligodendrogliomas were analyzed separately. In *IDH-*mut astrocytomas, residual tumor volume after surgery was an independent prognostic factor for survival (HR 1.02; 95% CI 1.01–1.03; p=0.003), but not in oligodendrogliomas (HR 1.02; 95% CI 1.00–1.03; p=0.15). Preoperative tumor size was an independent predictor in both astrocytomas (HR 1.03; 95% CI 1.00–1.05; p=0.02) and oligodendrogliomas (HR 1.05; 95% CI 1.01–1.09; p=0.01). Age was not a significant prognostic factor in multivariable analyses (astrocytomas p=0.64, oligodendrogliomas p=0.08).

**Conclusion:**

Our findings suggest that WHO grade is not a robust prognostic factor in molecularly well-defined dLGG. Preoperative tumor size remained a prognostic factor in both *IDH*-mut astrocytomas and oligodendrogliomas in our cohort, whereas residual tumor volume predicted prognosis in *IDH*-mut astrocytomas only. The age cutoffs for determining high risk in patients with *IDH*-mut dLGG from the pre-molecular era are not supported by our results.

## 1 Introduction

The advent of molecular markers in brain tumor classification, including diffuse low/lower-grade glioma (dLGG), has significantly improved prognostication of the clinical course (1–4). However, the currently used clinical prognostic factors were established in the pre-molecular era (5–9). The new 2021 WHO classification, based on a combination of molecular and histological tumor features, may influence the relevance of the earlier defined prognostic factors (10). This has led to questioning of the prognostic importance of WHO grade in dLGG harboring *isocitrate dehydrogenase*-mutation (*IDH*-mut), that is now considered a hallmark of this entity (1, 11, 12). For the *IDH*-mut tumors, the term “lower-grade glioma,” referring to WHO grade 2 and 3 tumors, has gained increased use after being coined by the TCGA group (1). However, the question remains whether WHO grade 2 and 3 diffuse glioma with *IDH*-mut can follow the same prognostic scoring models and thus should be viewed together, or still need to be addressed separately. Analyzing them as one group is in line with the current trend, where the term “lower-grade glioma” is more strongly linked to the *IDH* mutational status of the tumor than the WHO grade. A minority of patients with morphological lower-grade glioma have *IDH* wild-type (*IDH*-wt) tumors that in classifications before 2016 were considered together with *IDH*-mut tumors. It is now known that most of the *IDH*-wt dLGG show molecular resemblance to glioblastoma, and in the WHO 2021 classification these are indeed classified as such (10).

Of further relevance to the dLGG *IDH-*mut gliomas is the homozygous deletion of cyclin-dependent kinase inhibitor 2A/B (*CDKN2A/B*) that is associated with markedly shorter overall survival in *IDH*-mut tumors (13–18). For *IDH*-mut astrocytomas, the presence of *CDKN2A/B* homozygous deletion now classifies the tumor as a WHO grade 4 astrocytoma, even in the absence of histopathological features of necrosis and microvascular proliferation (10). Therefore, excluding *CDKN2A/B* homozygous deleted tumors captures a more homogenous group of *IDH*-mut dLGG, reflecting the current classification and inherent prognosis. Hence, there is an apparent need to re-evaluate earlier defined predictors of outcome, including WHO grade, in these tumors.

In this study, we hypothesized that the distinction between WHO grade 2 and 3 gliomas in a molecular well-defined cohort of *IDH*-mut tumors is of limited clinical relevance. If true, WHO grade 2 and 3 diffuse gliomas can be analyzed and studied together, with potential implications for the clinical management as well as for designs of clinical studies. Further, we aimed to evaluate the role of earlier well-established prognostic factors in the more homogenous molecular subclasses (5–7).

## 2 Materials and Methods

### 2.1 Study Population

The study population consists of patients with histopathologically verified WHO grade 2 or 3 diffuse gliomas in the period from 2007 through 2018, from a single center that serves all residents in a geographical defined catchment area which covers approximately 1.8 million inhabitants. Patients with a histological grade 2 or grade 3 glioma diagnosis derived from a biopsy only, in a tumor with radiological features highly significative of glioblastoma (ringlike contrast enhancement and necrosis), were not included in our institutional dLGG database, since sampling bias may play a significant role in these cases, thereby limiting the diagnostic accuracy (19, 20). The end of follow-up was December 1, 2020 for *IDH*-mut tumor patients and January 1, 2019 for *IDH*-wt tumor patients.

### 2.2 Clinical Variables

Data on patient age, sex, neurological condition, Karnofsky Performance Status (KPS), postoperative treatment, tumor size, tumor appearance, and location were retrieved from patient records and radiological imaging. Eloquence was assessed according to the definition from Chang and colleagues (6). “Early postoperative treatment” was defined as treatment within 6 months after surgery.

The volume of residual tumor after surgery was determined by tumor volume segmentation. The tumor volume was evaluated by semi-automatic segmentation performed with the open-source software “3DSlicer,” version 4.6.2 or newer (21). For the segmentation of tumor volume, we used the tools “LevelTracingEffect,” “WandEffect,” “DrawEffect,” and “PaintEffect” in the “Editor” module when appropriate. Tumor volumes were computed by the segmentation of hyperintense areas on the T2 or FLAIR sequence on MRI examinations. In exceptional cases, hyperintense areas were attributed to edema and therefore not included. Segmentation was performed by different trained personnel, but in all cases verified by a senior neurosurgeon (AJ) and with neuroradiological expertise consulted in selected cases. MRI examinations used in this project were performed with different MRI systems, including both 1.5T and 3.0T, and examinations can therefore have different echo, repetition, and inversion time. When available we used MRI with thin slices (typical 1 mm) and no interslice gap.

### 2.3 Histopathological Diagnosis and Molecular Data

The histopathological evaluation was made in accordance with the WHO criteria valid at the time of surgery and reclassified according to the WHO classification of 2021. Molecular analysis of *IDH*-mutation, 1p/19q codeletion, and homozygous deletion of *CDKN2A/B* were performed as previously described (22). Immunohistochemistry on formalin-fixed paraffin-embedded sections with antibodies against Ki67 to detect the fraction of proliferating cells in the tumor was performed as described earlier (23).

### 2.4 Statistics

All analyses were done with SPSS, version 26 or newer (Chicago, IL, USA). Statistical significance level was set to p<0.05. All tests were two-sided. Central tendencies are presented as means ± SD, or median with SE or first and third quartile if skewed. Dichotomous data were analyzed with Fisher Exact test. Overall survival was estimated by Kaplan-Meier method, and differences between groups were compared using log-rank test. In multivariable Cox regression analyses, only variables with a p-value ≤ 0.1 in the univariable analyses were included. In situations of multicollinearity between continuous variables, the significance level from the univariable analysis was used to select which of the variables that was used in the multivariable model presented in tabular form.

## 3 Results

### 3.1 WHO Grade and *IDH* Mutation

**Figure 1:**
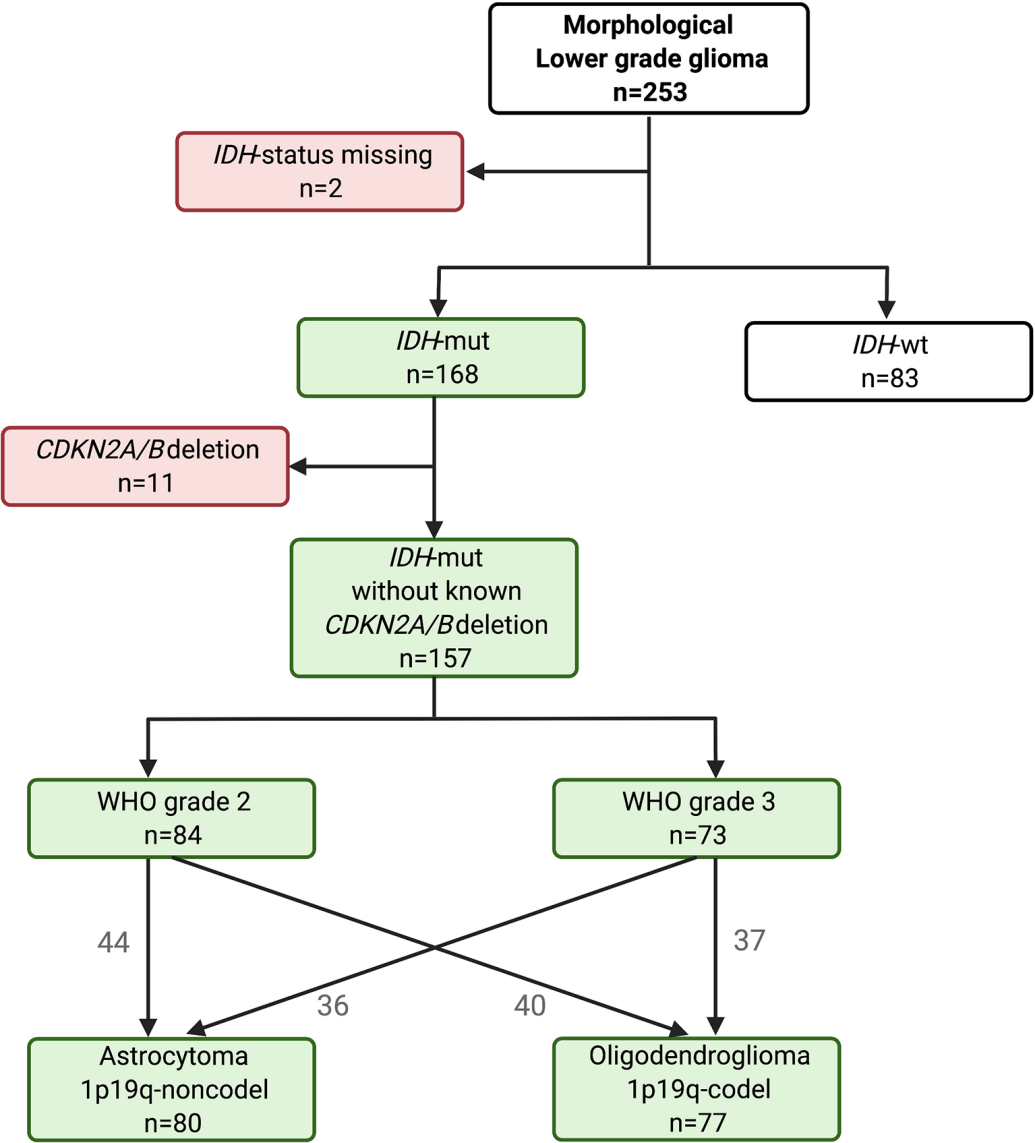
Distribution chart of *IDH* mutational status, and in the *IDH*-mut tumors, distribution of WHO grade and molecular subtype after omission of known *CDKN2A/B* homozygous deletion tumors.

The distribution of the 253 morphological dLGG over molecular subtypes is seen in **Figure 1**. The 83 patients (33%) with *IDH*-wt tumors were significantly older than those with *IDH*-mut tumors (p<0.00001) [median age 56 years (Q1/Q3:43/64) *vs* 41 years (Q1/Q3:33/53]. Patients with *IDH*-wt tumor had a shorter overall survival (log rank p<0.00001, **Supplementary Figure 1**).

### 3.2 *IDH*-Mutated dLGG

A separate assessment of the 168 *IDH*-mutated dLGG was made, with group level comparisons between the WHO grade 2 and grade 3 tumors (baseline characteristics and comparisons over grade presented in **Table 1**). The median follow-up (reversed Kaplan-Meier) for the *IDH*-mutated tumor patients (n=168) was 7.3 years (95% CI 6.7–7.8). Most variables were evenly distributed across grades, but contrast enhancement and the homozygous deletion of *CDKN2A/B* were overrepresented in WHO grade 3 tumors. Further, only 1% of patients (1/83) with WHO grade 3 tumors were asymptomatic, in contrast to 14% (12/85) of patients with WHO grade 2 tumors. As expected, Ki-67 labeling index was higher in tumors with higher WHO grade. Finally, it was more common for the WHO grade 3 patients to receive both early and late adjuvant therapy.

**Table 1:** Tumor and patient characteristics in *IDH*-mut dLGG by WHO grade.

	All dLGG *IDH*-mut	WHO grade 2 dLGG *IDH*-mut	WHO grade 3 dLGG *IDH*-mut	p-value*
	(n = 168)	(n = 85)	(n = 83)	
**Age, median (Q1-Q3)**	41 (33.0–52.8)	42 (33.5–52.5)	40 (32.0–53.0)	0.26
**Female, n (%)**	69 (41.1)	35 (41.2)	34 (41.0)	0.98
**KPS** ≤ **80**	66 (39.3)	29 (34.1)	37 (44.6)	0.21
**Focal deficit, n (%)**	28 (16.7)	14(16.5)	14 (16.9)	1.00
**Asymptomatic/incidental finding, n (%)**	13 (7.7)	12 (14.1)	1 (1.2)	**0.002**
**Max tumor diameter in mm, mean (SD)**	56.3 (19.4)	55.2 (19.6)	57.3 (19.3)	0.48
**Eloquence, n (%)**	111 (66.1)	59 (69.4)	52 (62.7)	0.43
**Any CE, n (%)**	81 (48.8)	28 (33.3)	53 (64.6)	**<0.001**
missing	n=2	n=1	n=1	
**Bilateral growth, n (%)**	19 (11.3)	8 (9.4)	11 (13.3)	0.43
**Tumor border, n (%)**				1.0
absent	20 (12.0)	10 (11.8)	10 (12.3)	
conspicuous or mild	146 (88.0)	75 (88.2)	71 (87.7)	
missing	n=2		n=2	
**Mainly frontal location, n (%)**	104 (61.9)	51 (60.0)	53 (63.9)	0.64
**1p19q-codeletion, n (%)**	79 (46.4)	41 (48.2)	38 (44.6)	0.76
**Ki-67%, mean (SD)**	2.5 (3.6)	1.7 (2.2)	3.3 (4.3)	**0.007**
missing	n=45	n=29	n=16	
**Homozygous loss of *CDKN2A/B*, n (%)**	11 (9.3)	1 (1.9)	10 (15.2)	**0.022**
missing	n=50	n=33	n=17	
**Biopsy only, n (%)**	9 (5.4)	4 (4.8)	5 (6.1)	0.74
**Residual volume ml, median (Q1-Q3)**	8.9 (1.4–25.4)	9.1 (1.3–22.3)	8.9 (1.6–31.8)	0.51
Early postoperative chemotherapy**	89 (53.9)	29 (34.1)	60 (75.0)	**<0.0001**
missing	n=3		n=3	
**Any postoperative chemotherapy**	135 (82.3)	62 (73.8)	73 (91.3)	**0.004**
missing	n=4	n=1	n=3	
Early postoperative radiotherapy**	98 (58.7)	32 (37.6)	66 (80.5)	**<0.0001**
missing	n=1		n=1	
**Any postoperative radiotherapy**	139 (84.2)	64 (77.1)	75 (91.5)	**0.02**
missing	n=3	n=2	n=1	
Early postoperative chemo- or radiotherapy**	119 (71.3)	41 (48.2)	78 (95.1)	**<0.0001**
missing	n=1		n=1	
Early postoperative radio-chemotherapy (both)**	68 (41.2)	20 (23.5)	48 (60.0)	**<0.0001**
missing	n=3		n=3	
**Deceased, n (%)**	57 (33.9)	26 (30.6)	31 (37.3)	0.41
**Survival years, median (95%CI)**	10.2 (8.5–11.9)	11.4 (8.7–14.1)	10.0 (7.4–12.6)	0.54

KPS, Karnofsky Performance Status; CE, contrast enhancement.

*Comparing WHO grade 2 and WHO grade 3.

**Early therapy was defined as treatment within 6 months after surgery. Bold text indicates p-value <0.05.

### 3.3 *IDH*-Mutated dLGG Without Known Homozygous Deletion of *CDKN2A/B*

*CDKN2A/B* status was available in only 118 of the 168 *IDH*-mut gliomas. Of these, 11 had *CDKN2A/B* homozygous deletions. Among the *CDKN2A/B* deleted tumors, all but two were astrocytomas grade 3. Two were oligodendrogliomas (WHO grade 2 and WHO grade 3). Clinical characteristics of these patients are presented in **Supplementary Table 1**. Patients with *CDKN2A/B* homozygous deleted tumors had a worse prognosis than patients without deletion (p= 0.0002, **Supplementary Figure 2**). For all further analyses in the *IDH*-mut group, these 11 *CDKN2A/B* homozygous deleted tumors were excluded (**Figure 1**).

### 3.4 WHO Grade

**Figure 2:**
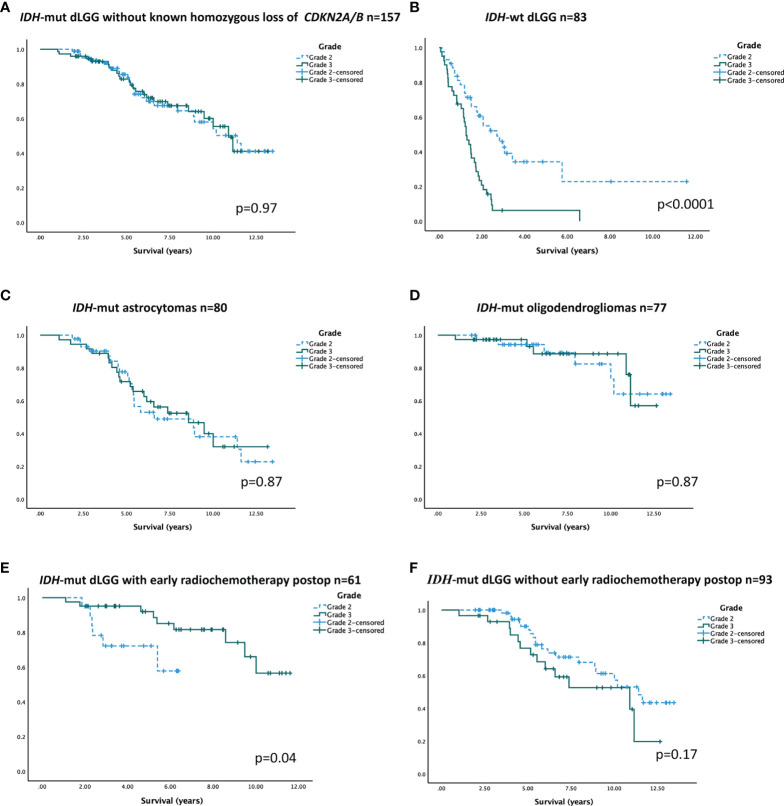
Overall survival over WHO grade in different patient subgroups **(A–F)**. **(A)** *IDH*-mut dLGG (grade 2; 11.4 years; 95% CI 8.7–14.1 and grade 3; 10.9 years; 95% CI 9.5–12.3). **(B)** *IDH*-wt dLGG (grade 2; 2.7 years; 95% CI 1.7–4.7 and grade 3; 1.3 years; 95% CI 1.0–1.5). **(C)** *IDH*-mut astrocytoma (grade 2; 6.6 years; 95% CI 1.8–11.4 and grade 3; 8.6 years; 95% CI 5.2–11.9). **(D)** Oligodendroglioma (median survival not reached). **(E)** *IDH*-mut patients receiving radio-chemotherapy within 6 months postoperative (median survival not reached). **(F)** *IDH*-mut tumor patients not receiving radio-chemotherapy within 6 months postoperative (grade 2; 11.4 years; 95% CI 9.3–13.5 and grade 3; 10.9 years; 95% CI 4.9–16.9). In all figures showing *IDH*-mut tumors, tumors with known *CDKN2A/B* homozygous deletions were omitted.

When analyzing survival in the cohort of *IDH*-mutated tumors without known *CDKN2A/B* homozygous deletions, survival curves for WHO grade 2 and 3 tumors overlapped. Median overall survival in WHO grade 2 tumors was 11.4 years (95% CI 8.7–14.1) compared to 10.9 years (95% CI 9.5–12.3) in WHO grade 3 tumors (log rank test p=0.97, **Figure 2A**). In contrast, for *IDH*-wt tumors, WHO grade was a strong prognostic factor (log rank test p< 0.0001, **Figure 2B**). In *IDH*-mutated tumor subtypes without known *CDKN2A/B* homozygous deletions, there was no significant difference in overall survival between WHO grade 2 and 3 (**Figures 2C, D**).

To address the difference in postoperative treatment administered for *IDH*-mut WHO grade 2 and 3 tumors, respectively, and its potential impact on overall survival, a sensitivity analysis was carried out in treatment-homogenous strata. In the stratum of patients treated with early radio-chemotherapy, patients with WHO grade 2 tumors (n=19) had a shorter survival than patients with WHO grade 3 tumors (n=41) (**Figure 2E**). In patients not receiving early radio-chemotherapy (n=93), there was no significant difference in survival between WHO grade 2 and 3 tumor patients (**Figure 2F**).

A sensitivity analysis including only *IDH*-mut tumors that were confirmed to lack *CDKN2A/B* homozygous deletion (n=107) confirmed that there was no difference in survival between patients with WHO grade 2 and grade 3 dLGG (p=0.60) (**Supplementary Figure 3**). Astrocytomas and oligodendrogliomas were also assessed separately, showing results consistent with those in the main analysis (**Supplementary Figure 3**).

### 3.5 Prognostic Factors

We performed Cox regression analyses using variables with potential prognostic value for astrocytoma and oligodendroglioma, respectively. Variables with a p-value ≤ 0.1 in the univariable analyses were included in the multivariable models. Hazard ratio (HR) with confidence interval and p-values for the different variables are presented in **Table 2**.

**Table 2:** Univariable and multivariable Cox regression analysis for survival in *IDH*-mut lower grade astrocytomas (1p19q-noncodel) and oligodendrogliomas (1p19q-codel) without known *CDKN2A/B* homozygous deletion, n=157.

Variable	Univariable HR (95% CI)	p-value	Multivariable HR (95% CI)	p-value
**Male sex (ref female)**				
Astrocytoma	1.35 (0.70–2.59)	0.38		
Oligodendroglioma	0.58 (0.17–1.97)	0.38		
**Age (years)**				
Astrocytoma	1.03 (1.00–1.05)	**0.038**	1.01 (0.98–1.04	0.64
Oligodendroglioma	1.06 (1.01–1.10)	**0.025**	1.05 (1.00–1.10)	0.079
**Asymptomatic (ref symptomatic)**				
Astrocytoma	1.44 (0.34–6.00)	0.62		
Oligodendroglioma	0.48 (0.06–4.11)	0.50		
**KPS score 80 or below (ref KPS 90-100)**				
Astrocytoma	1.92 (1.02–3.63)	**0.044**	1.79 (0.83–3.84)	0.14
Oligodendroglioma	1.45 (0.42–4.97)	0.56		
**Max tumor diameter (mm)**				
Astrocytoma	1.04 (1.02–1.05)	**<0.00001**		
Oligodendroglioma	1.05 (1.02–1.08)	**0.001**	1.05 (1.01–1.09)	**0.01**
**Tumor location non-frontal (ref frontal)**				
Astrocytoma	1.87 (0.63–2.23)	0.60		
Oligodendroglioma	0.35 (0.04–2.72)	0.31		
**Eloquence (Chang) (ref non-eloquent)**				
Astrocytoma	2.87 (1.12–7.36)	**0.028**	1.72 (0.62–4.74)	.30
Oligodendroglioma	0.44 (0.13–1.56)	0.21		
**Bilateral growth (ref unilateral)**				
Astrocytoma	2.56 (1.06–6.17)	**0.037**	0.42 (0.09–1.88)	.26
Oligodendroglioma	2.09 (0.44–10.01)	0.38		
**Lack of tumor border (ref detectable tumor border)**				
Astrocytoma	4.48 (1.99–10.11)	**<0.001**	1.33 (0.33–5.39)	.69
Oligodendroglioma	2.64 (0.68–10.29)	0.16		
**Contrast enhancement (ref no enhancement)**				
Astrocytoma	1.51 (0.79–2.87)	0.21		
Oligodendroglioma	1.22 (0.34–4.37)	0.76		
**Focal deficit (ref no focal deficit)**				
Astrocytoma	1.72 (0.78–3.77)	.18		
Oligodendroglioma	6.48 (1.54–27.34)	**.01**	2.21 (0.49–10.11)	.30
**Grade 3 (ref grade 2)**				
Astrocytoma	0.95 (0.50–1.79)	0.87		
Oligodendroglioma	0.91 (0.28–2.99)	0.88		
**Early postop radio-chemotherapy (within 6 months) (ref no early R-C-therapy)**				
Astrocytoma	0.77 (0.39–1.51)	0.44		
Oligodendroglioma	0.32 (0.04–2.50)	0.28		
**Residual tumor volume above 10 ml (ref volume ≤ 10 ml)**				
Astrocytoma	5.90 (2.69–12.92)	**<0.00001**	[6.30 (2.22–17.86)]*	(0.001)*
Oligodendroglioma	1.60 (0.43–5.98)	0.48		
**Residual tumor volume (ml)**				
Astrocytoma	1.02 (1.01–1.03)	**<0.000001**	1.02 (1.01–1.03)	**0.003**
Oligodendroglioma	1.01 (1.00–1.03)	0.085		

KPS denotes Karnofsky Performance Status.

*Results from a separate multivariable analysis where the continuous variable of residual tumor volume is replaced by a dichotomized counterpart. Two additional multivariable models are presented in the text but not shown in this table for clarity.

Bold text indicates p-value < 0.05.

To avoid multicollinearity, the significantly correlated variables preoperative “maximal tumor diameter” and postoperative “residual tumor volume” were not used in the same models.

#### 3.5.1 Astrocytomas

For *IDH*-mut astrocytomas (n=80), residual tumor volume remained a significant predictor for survival (HR 1.02; 95% CI 1.01–1.03; p=0.003) after adjusting for the effects of age, tumor border, bilateral tumor growth, eloquent tumor location, and performance status. An additional multivariable model was made with preoperative maximal tumor diameter instead of residual volume (not presented in the table). In this analysis, tumor diameter was the only significant prognostic factor for survival (HR 1.03; 95% CI 1.00–1.05; p=0.02).

We performed two sensitivity analyses including only *IDH*-mut astrocytomas verified to lack *CDKN2A/B* homozygous deletion (n=55). We included the same selection of variables as above. In the model with residual tumor volume, this was again the only independent prognostic factor (HR 1.03; 95% CI 1.01–1.05; p<0.001). In the model with maximal tumor diameter instead of residual tumor volume, the HR of maximal tumor diameter was similar to that of the main analysis but no longer statistically significant (HR 1.02; 95% CI 0.99–1.05; p<0.14), and neither was HR for any of the other variables (p-value range between 0.16 and 0.95).

#### 3.5.2 Oligodendrogliomas

Multivariable analysis in oligodendrogliomas (n=77) with preoperative maximal tumor diameter, age, and focal deficit showed that maximal tumor diameter remained significantly associated with survival (HR 1.05; 95% CI 1.01–1.09: p=0.01). Age was not significantly associated with survival in the multivariable analysis (HR 1.05; 95% CI 1.00–1.10; p=0.079). A second multivariable model using “residual tumor volume” instead of “maximal tumor diameter” was carried out for oligodendrogliomas (not presented in table). In this model, only focal deficit was associated with survival (HR 6.43; 95% CI 1.26–32.72; p=0.025). Neither age (HR1.03; 95% CI 0.97–1.10; p=0.36) nor residual tumor volume (HR 1.02; 95% CI 1.00–1.03; p=0.15) was associated with survival.

**Figure 3:**
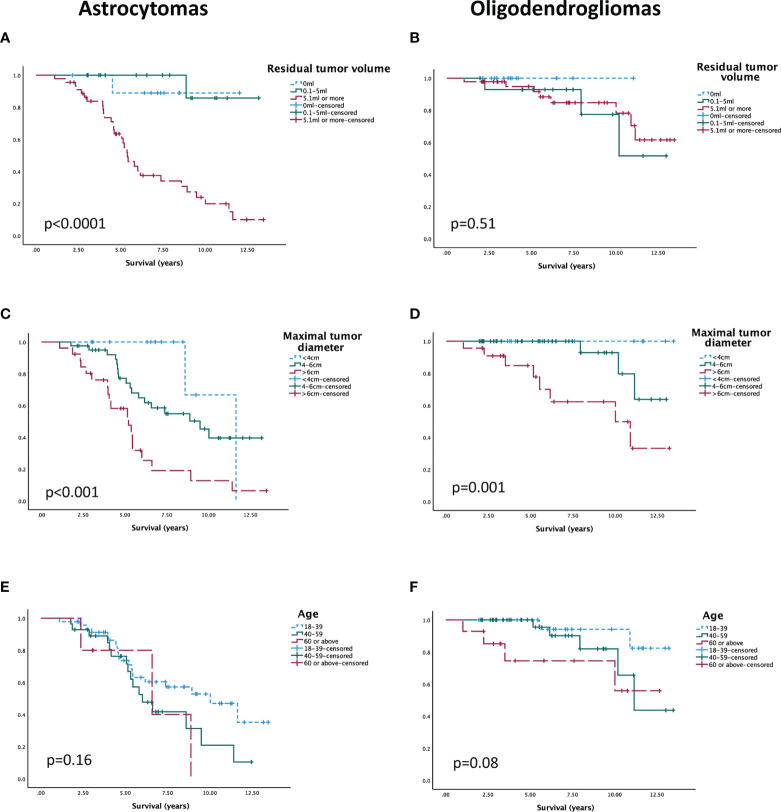
Unadjusted survival over “residual tumor volume” in **(A)** astrocytomas and **(B)** oligodendrogliomas, over “maximal tumor diameter” in **(C)** astrocytomas and **(D)** oligodendrogliomas, and over “age” in **(E)** astrocytomas and **(F)** oligodendrogliomas.

For graphic illustration, groups were made for residual tumor volume, maximal preoperative diameter, and patient age and presented in unadjusted Kaplan-Meier curves for astrocytomas and oligodendrogliomas, respectively (**Figures 3A–F**), whereas survival times are summarized in **Supplementary Table 2**.

#### 3.5.3 Non-Subgroup Analysis

When *IDH*-mutated astrocytomas and oligodendrogliomas without known *CDKN2A/B* homozygous deletion were analyzed together (n=157), age did not significantly affect the hazard ratio for overall survival in univariable analysis (p=0.12). Median age differed between the subtypes, with 35.5 years (Q1:Q3; 30:50) for astrocytoma patients and 45 years (Q1:Q3; 38.5:56.5) for oligodendroglioma patients (p<0.001).

## 4 Discussion

In this population-based study we found that WHO grade did not carry prognostic relevance in a molecularly well-defined cohort of *IDH*-mutated dLGG, regardless of molecular subtype. We could also confirm the prognostic importance of *CDKN2A/B*.

Of the previously recognized clinical prognostic factors, we found that preoperative and postoperative tumor burden were significant predictors for survival in *IDH*-mutated astrocytomas. In fact, the preoperative tumor burden was the only factor consistently associated with survival for oligodendrogliomas in the cohort.

### 4.1 WHO Grade

WHO grade was not prognostic for survival in *IDH*-mutated dLGG cleared from tumors with *CDKN2A/B* homozygous deletion. The finding was consistent across the different subtypes, when analyzed separately. Our results are in accordance with several earlier studies pointing to a lack of prognostic impact of WHO grade in molecularly subtyped *IDH*-mutated dLGG (1, 2, 11, 15, 24–29), including the absence of prognostic relevance among tumors with *CDKN2A/B* homozygous deletion (14). Shirahata et al. reported a loss of significant difference in OS between WHO grade 2 and grade 3 *IDH*-mutated astrocytomas when tumors with *CDKN2A/B* deletions were removed (18).

Yet other studies have shown WHO grade to be a prognostic marker in *IDH*-mutated dLGG, at least for astrocytomas (30–32). However, in none of these studies, tumors with *CDKN2A/B* homozygous deletion were excluded. The prognostically unfavorable *CDKN2A/B* homozygous deletion is more common in grade 3 tumors (and especially in astrocytomas), which was found to be the case also in our material and which may contribute to a shorter survival in cohorts of WHO grade 3 tumors where this deletion has not been adjusted for (14, 18, 33, 34).

Another explanation contributing to the varying results may be the well-described inter-observer variability and different practices in classifying a tumor as WHO grade 2 or WHO grade 3, partly due to a lack of defined mitotic threshold (35, 36). This lack of robustness may in itself be a reason to rely less on WHO grade as a predictor for prognosis and in clinical decision-making in *IDH*-mutated dLGG.

As emphasized by several authors, it is relevant to consider the impact of chemo- and radiotherapy when retrospectively assessing prognostication (30, 37). In many institutions, upfront treatment is standard of care in tumors of WHO grade 3 but administered only to a selection of patients with WHO grade 2 tumors. While it is reasonable to believe that the decision to withhold treatment for WHO grade 2 tumors is often due to a prognostically favorable situation, the deviation from postoperative treatment in WHO grade 3 tumors on the other hand may reflect prognostically unfavorable factors such as compromised general condition of the patient or tumor volume considered too large to irradiate. This is well in line with our results, showing tendencies for worse survival in patients with WHO grade 3 in the non-treated stratum but significantly worse outcome for grade 2 tumor patients in the stratum of promptly treated patients. Similar observations were made in another recent retrospective study of treatment impact, comparing adjuvantly treated and untreated WHO grade 2 diffuse gliomas after surgery, with worse outcome in the group receiving adjuvant therapy (38). Other retrospective studies have shown more favorable outcome in patients having undergone radio-chemotherapy or radiotherapy, but it is difficult to draw firm conclusions on treatment effect by this method (30, 31). Overall, we must bear in mind the possibility both of selection bias being responsible for differences in outcome for different treatment groups and a lack of difference in survival between dLGG WHO grade 2 and WHO grade 3 being attributable to treatment effects.

### 4.2 Prognostic Factors

In this study we used a wide range of prognostic factors from the pre-molecular era and analyzed their relevance in our more homogenous cohort (5–7). To our knowledge, there is no other study evaluating these factors after exclusion of patients with known *CDKN2A/B* deletions. First of all, we could confirm the adverse prognostic effect of *CDKN2A/B* homozygous deletion in *IDH*-mut dLGG. Although the number of patients with detected deletion in our cohort was too small for meaningful statistics, the impression was that they show many characteristics that are associated with unfavorable outcome in unselected dLGG (large size before and after surgery, heavy burden of symptoms, contrast enhancement, older age and high Ki-67-index).

#### 4.2.1 Residual Tumor Volume

Increased extent of resection (EOR) has been identified as a predictor for prolonged survival in *IDH*-mutated dLGG (39–43). Many studies have not distinguished between astrocytomas and oligodendrogliomas, but when done, the favorable effect of extensive resection is often more evident in astrocytomas (32, 41, 44). This was also the case in our material, where decreased residual tumor volume was an independent predictor for prolonged survival in astrocytomas but not in oligodendrogliomas.

However, like most studies on the topic, the proportion of events in oligodendrogliomas was low (14.3%) in our cohort, making robust survival analysis difficult to achieve in this subgroup.

#### 4.2.2 Tumor Size

Tumor size is an important prognostic factor from the pre-molecular era that retained its role as an independent predictor for survival in both *IDH*-mutated astrocytomas and oligodendrogliomas. Preoperative tumor size was not an independent significant prognostic factor in one sensitivity analysis. Even though this may call the robustness of the finding into question, it is highly plausible that it is related to the limited power of this sub-analysis. Overall, the findings are in accordance with earlier observations, even though *IDH*-mutated astrocytomas and oligodendrogliomas have not always been presented separately (41, 43, 45). Apart from the intuitive and observed correlation between preoperative and postoperative tumor size (41, 46) as well as the apparent linkage to other unfavorable factors such as multilobar growth, eloquence, performance status, and neurological deficits, there may be inherent biological adverse effects from large tumor size per se. This concept is to some degree supported by a recent, albeit small, study observing that genomic instability correlated with larger size in *IDH*-mutated astrocytomas (47).

#### 4.2.3 Age

Our results give no convincing support to the much used 40-year age limit for high risk, a cutoff presumably confounded by the presence of lower-grade *IDH*-wt tumors (8, 9). In our study, the prognostic role of age was less prominent, especially for *IDH*-mut astrocytomas. The results are partly in line with findings in other studies where patient age showed no significant effect on survival in *IDH*-mut astrocytomas (11, 24, 26), but in *IDH*-mut oligodendrogliomas (24). In another publication, significant correlations were seen between age and survival in both *IDH*-mut tumor subtypes (29). A recent study exploring age in patients with dLGG found an association between worse survival and increasing age in the *IDH*-wt group only, an observation also reported earlier (27, 48). However, in these studies, oligodendrogliomas and astrocytomas were analyzed together, and the younger median age in patients with astrocytomas may confound a potential age effect in oligodendrogliomas, and *vice versa*. To draw reliable conclusions about age and dLGG, subgroups need to be analyzed separately.

### 4.3 Strengths and Limitations

To our knowledge, this is the first study to assess prognostic factors in the clinically relevant entities of *IDH*-mutated astrocytoma and oligodendroglioma, respectively, without known *CDKN2A/B* homozygous deletions. There are, however, limitations to the study, based on the retrospective design with its inherent risk for non-causal associations and, as already mentioned, with difficulties in separating treatment effects from natural course of disease. Many variables are interlinked, and awareness of multicollinearity is necessary, even though we have sought to minimize this through restrictiveness in including covariates for multivariable analysis. Just like in most studies with individual level data on subcategorized tumors, the cohort sizes are rather small, which limits some of the subgroup analyses. Also, a limited follow-up time of 7.3 years may be too short, especially in patients with oligodendroglioma. Further, since a portion of the patients were diagnosed in the pre-molecular era, molecular analyses have been performed retrospectively. For 50 cases, *CDKN2A/B* status was not established, a limitation that partly be remedied through sensitivity analyses including only tumors with known *CDKN2A/B* status. Strengths of the study include the population-based setting, the detailed clinical and treatment data, the volumetric assessment of residual tumor volume, and molecularly defined subgroups analyzed separately.

## 5 Conclusion

In molecularly well-defined dLGG, WHO grade does not seem to be a reliable stratifier for risk. Our results suggest that residual tumor volume remains a major prognostic factor for astrocytomas, while preoperative tumor size is prognostic in both *IDH*-mutated astrocytomas and oligodendrogliomas. The prognostic role of age, on the other hand, appears to be weaker than in the pre-molecular era. We can conclude that especially for astrocytomas, previous findings pertaining to age are likely biased by the inclusion of *IDH*-wt tumors.

## Data Availability Statement

The raw data supporting the conclusions of this article will be made available by the authors, without undue reservation.

## Ethics Statement

The studies involving human participants were reviewed and approved by the regional ethical committee in the region of Västra Götaland (DNR 1067-16). The need for informed consent prior to 2017 was waived by the ethical committee and following 2017 it was based upon written informed consent.

## Author Contributions

LC: conception and design, writing of draft, revising, editing, and submitting. AC: data collection, revising of manuscript, final approval. AS: supervision, conception and design, revising of manuscript, final approval. AD: data collection, revising of manuscript, final approval. HS: data collection, revising of manuscript, final approval. HC: data collection, revising of manuscript, final approval. HB: data collection, revising of manuscript, final approval. KM: data collection, revising of manuscript, final approval. SFV: data collection, revising of manuscript, final approval. TB: data collection, revising of manuscript, final approval. AJ: supervision, conception and design, writing of draft, revising, editing and financial support. All authors contributed to the article and approved the submitted version.

## Funding

This study received financial support in the form of ALF-grant (ALFGBG-716671) and funding from the Swedish Research Council (2017-00944), as well as grants from the Göteborg Medical Society (Göteborgs Läkaresällskap (GLS-960642)). The sponsors had no role in the design or conduct of this research.

## Conflict of Interest

AJ has received honoraria for education content from INOMED.

The remaining authors declare that the research was conducted in the absence of any commercial or financial relationships that could be construed as a potential conflict of interest.

## Publisher’s Note

All claims expressed in this article are solely those of the authors and do not necessarily represent those of their affiliated organizations, or those of the publisher, the editors and the reviewers. Any product that may be evaluated in this article, or claim that may be made by its manufacturer, is not guaranteed or endorsed by the publisher.
