# Supplementary material for: WHO Grade Loses Its Prognostic Value in Molecularly Defined Diffuse Lower-Grade Gliomas
Source: Front Oncol. 2022 Jan 10;11:803975. doi: 10.3389/fonc.2021.803975 (PMC8785215; doi:10.3389/fonc.2021.803975)
Supplement: Supplementary file 1 [file Table_1.docx]

**Supplementary table 1**.

Tumor and patient characteristics in *CDKN2A/B* homozygous deleted *IDH*-mut dLGG.

|  | Known CDKN2A/B homozygous deletion  n=11 | All other *IDH*-mut  lower-grade glioma  n=157 | p-value |
| --- | --- | --- | --- |
| Age, median (Q1/Q3) | 45 (40.0/58.0) | 41 (32.0/52.5) | 0.23 |
| Female sex, n (%) | 6 (54.5) | 63 (40 .1) | 0.36 |
| KPS ≤80, n (%) | 6 (54.5) | 60 (38.2) | 0.34 |
| Focal deficit | 4 (36.4) | 24 (15.3) | 0.09 |
| Asymptomatic/incidental finding, n (%) | 0 (0) | 13 (8,3) | 1.00 |
| Maximal diameter >6cm n, (%) | 5 (45.5) | 49 (31.2) | 0.33 |
| Tumor location mainly frontal n (%) | 7(63.6) | 97 (61.8) | 1.00 |
| Max tumor diameter, mm, mean (SD) | 60.0 (16.6) | 56.0 (19.6) | 0.31 |
| Contrast enhancement | 9 (81.8) | 72 (46.5) | **0.03** |
| Biopsy only | 1 (9.1) | 8 (5.1) | 0.47 |
| Residual volume ml, median (Q1-Q3) | 21.6 (4.2-65.2) | 8.8 (1.3-22.9) | 0.12 |
| Ki67%, mean (SD)  missing | 5.8 (8.4) | 2.2 (2.6)  45 | 0.19 |
| Deceased | 7 (63.6) | 50 (31.8) | **0.046** |
| Survival years, median (95% CI) | 5.2 (4.1- 6.4) | 11.1 (9.8-12.5) | **0.002** |

KPS denotes Karnofsky Performance Status
