# Supplementary material for: WHO Grade Loses Its Prognostic Value in Molecularly Defined Diffuse Lower-Grade Gliomas
Source: Front Oncol. 2022 Jan 10;11:803975. doi: 10.3389/fonc.2021.803975 (PMC8785215; doi:10.3389/fonc.2021.803975)
Supplement: Supplementary file 2 [file Table_2.docx]

| **Supplementary Table 2**  Unadjusted median survival for IDH-mut astrocytomas and oligodendrogliomas over "residual tumor volume", "maximal tumor diameter" and "patient age". | | | | |
| --- | --- | --- | --- | --- |
|  | Astrocytomas unadjusted  median survival; 95%CI | Log rank p-value  Astrocytoma | Oligodendrogliomas unadjusted  median survival; 95%CI | Log rank p-value  Oligodendroglioma |
| Residual tumor volume  0 ml  0.1-5ml  ≥5.1ml | not reached  not reached  5.4 years; 4.7-6.1 | p<0.0001 | not reached  not reached  not reached | p=0.51 |
| Maximal tumor diameter  <4cm  4-6cm  >6cm | 11.6 years; *  9.5 years; 6.1-12.9  5.2 years; 3.6-6.8 | p<0.001 | not reached  not reached  10.0 years; 5.2-14.8 | p=0.001 |
| Age  18-39  40-59  ≥60 | 10.0 years; 6.5-13.6  6.0 years; 4.4-7.6  6.6 years; 0.4-12.7 | p=0.16 | not reached  11.1 years; 9.3-13.0  not reached | p=0.08 |

* CI could not be calculated
