# Supplementary figures and images for: WHO Grade Loses Its Prognostic Value in Molecularly Defined Diffuse Lower-Grade Gliomas
Source: Front Oncol. 2022 Jan 10;11:803975. doi: 10.3389/fonc.2021.803975 (PMC8785215; doi:10.3389/fonc.2021.803975)

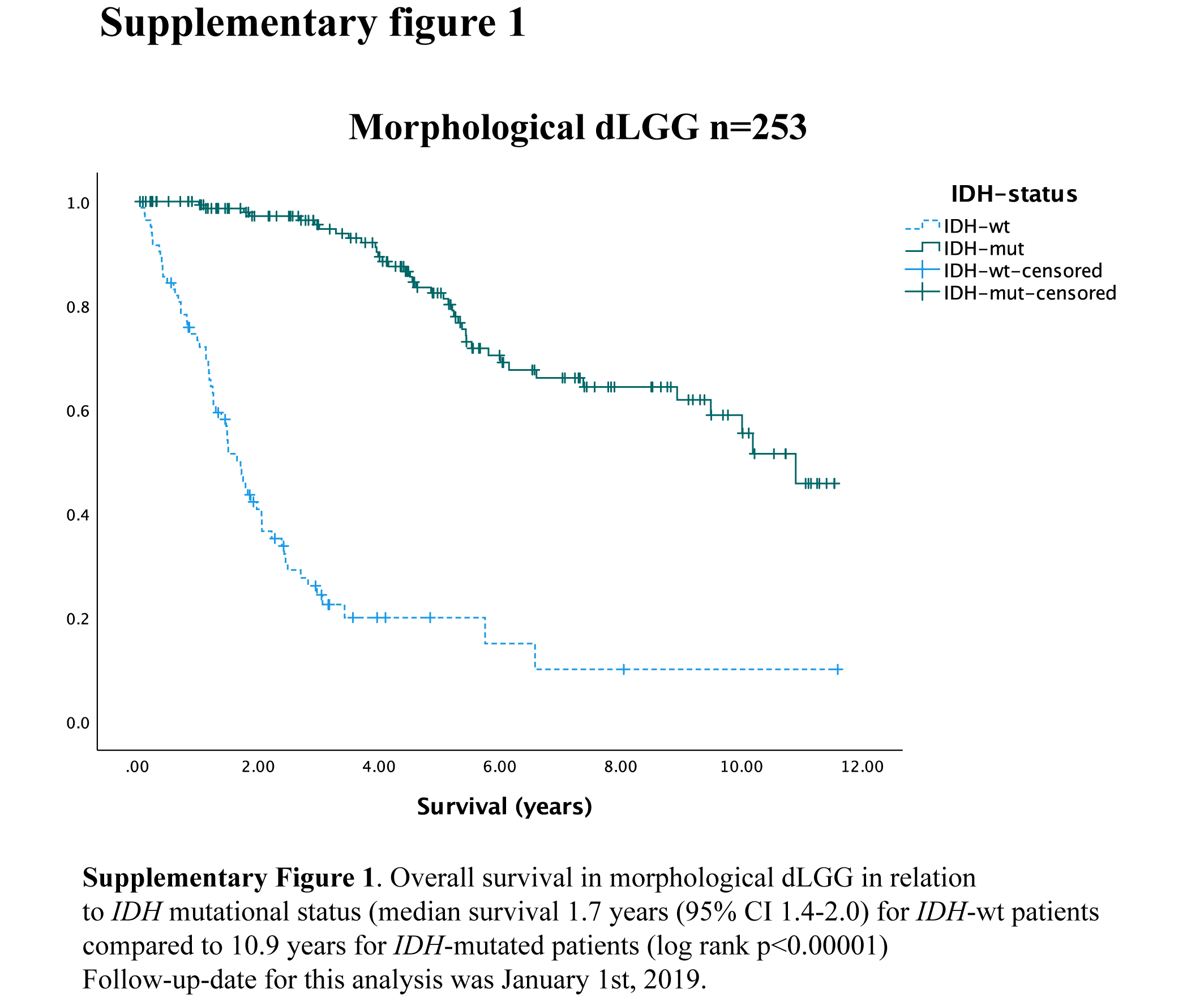

Supplement: Supplementary file 3 [file Image_1.tif]

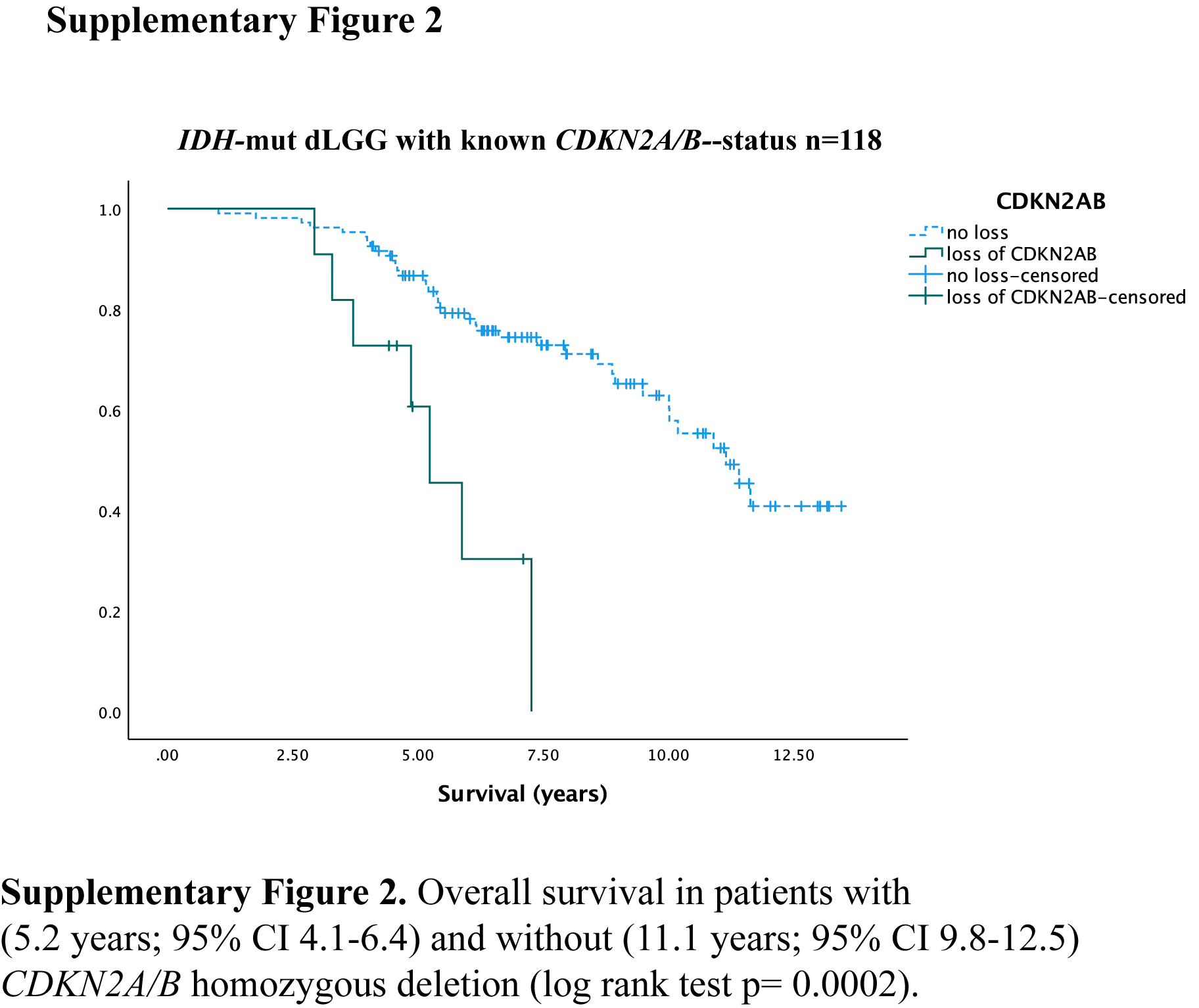

Supplement: Supplementary file 4 [file Image_2.tif]

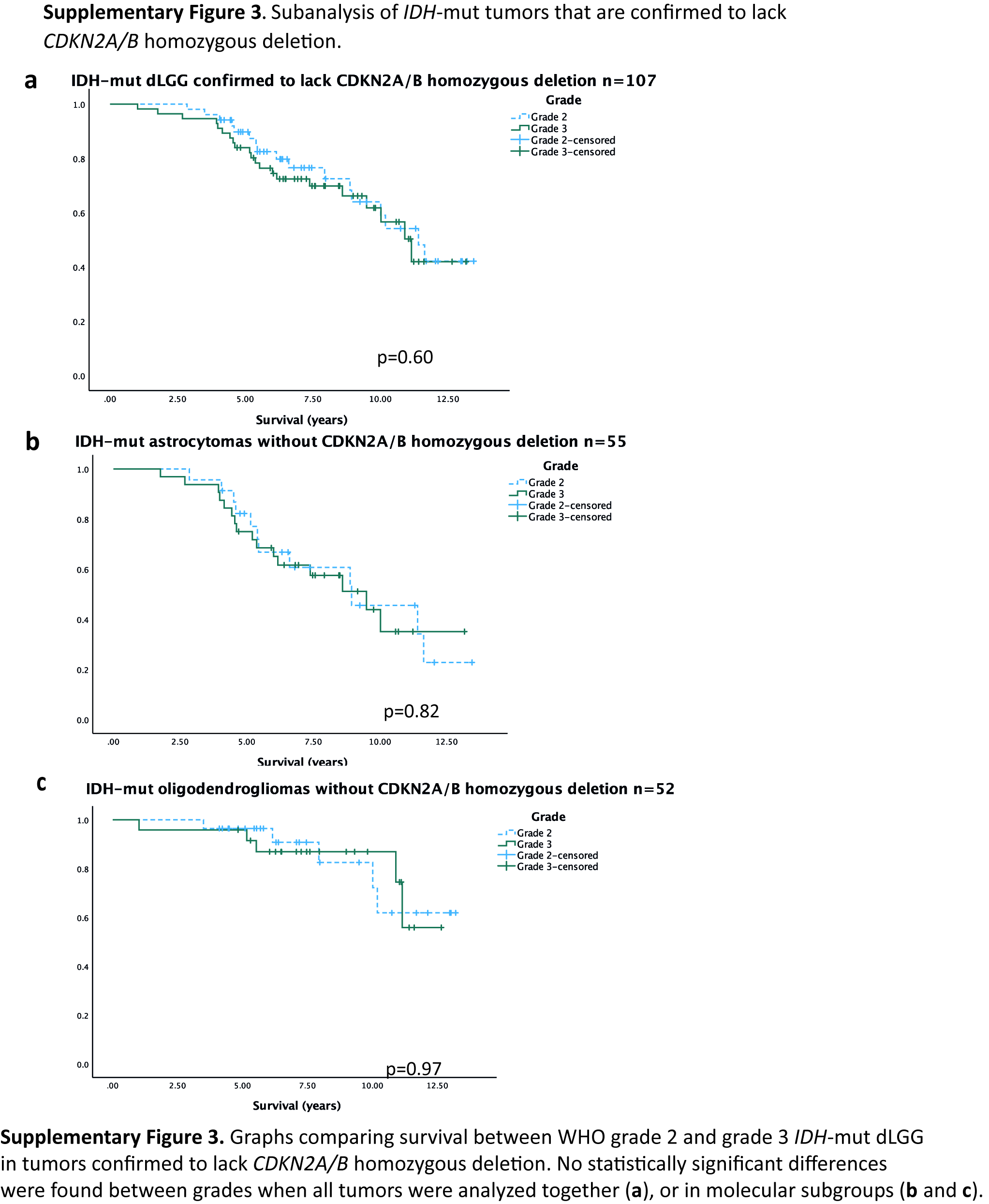

Supplement: Supplementary file 5 [file Image_3.tif]
